# Editorial Notes

**Published:** 1939

**Authors:** 


### Editorial Notes

**Bristol Royal Hospital.**

The Board of Charity Commissioners for England and Wales by an Order dated 15th September, 1939, approved and established a scheme whereby the Bristol Royal Infirmary and Bristol General Hospital shall be administered and managed as one Charity, under the title of the Bristol Royal Hospital, by a Board of Management to be known as the Hospital Board. Under this Scheme the endowments and funds of the two charities are transferred (with certain specified exceptions) to the "Official Trustees of Charitable Funds." The Hospital Board when complete will consist of :—

1. *Ex-officio Members* :—

The President of the Charity. The Vice-Presidents. The Honorary Treasurer. The Dean of the Faculty of Medicine.

2. *Co-optative Members* (not less than fourteen) :—

The following were named in the Scheme to hold office for one year and thereafter to comprise eight members of the House Committee of the Bristol Royal Infirmary and six members of the House Committee of the Bristol General Hospital. At least one Works Governor is to be included from each House Committee.

Lieut.-Col. P. G. Robinson, D.S.O., J.P. ; J. H. H. Perks, Esq. ; Walter Walters, Esq. ; J. A. Nixon, Esq. ; C.M.G., M.D. ; H. C. White, Esq. ; F. M. Burris, Esq. ; Clare Smith, Esq. ; H. M. Baker, Esq. ; Major Egbert Cadbury, D.F.C., D.S.C., J.P. ; C. Cyril Clarke, Esq. ; Lady (Vernon) Wills ; G. W. Anson, Esq. ; A. Esmond Robinson, Esq., M.C. ; Mrs. E. N. Harford.

3. *Nominated Members* (to hold office for one year) :—

One by the Senate of the University of Bristol. Three by Committees administering Hospital Contributory Schemes approved by the Hospital Board.

4. *Medical Members* :—

Six Medical Members appointed by the Honorary Medical Staff of the Charity.

Any member of the Hospital Board may be reappointed. The Honorary Medical Staff will be appointed by an *Election Committee* to consist of :—

The Members of the Hospital Board (including the Medical Members). Six Governors selected annually by the Governors at their Annual Meeting. Four Medical Committee men selected annually by the Honorary Medical Staff from their own number.

Thus the amalgamation of the two principal Voluntary Hospitals is completed. At present the two institutions will continue their work as heretofore in separate buildings, each with its own House Committee responsible to the Hospital Board. The Honorary Medical Staffs are merged into a single Staff. The Secretariats and the Nursing Staffs remain for the present independent of each other, excepting in so far as the General Management of the Charity is exercised by the Hospital Board.

**Bristol Hospitals Fund.**

THE Bristol Hospitals Fund has been started by the Bristol Royal Hospital, Bristol Royal Hospital for Sick Children and Women, Bristol Eye Hospital, Bristol Homœopathic Hospital and Cossham Memorial Hospital as their official Contributory Scheme.

In the opinion of the hospitals the establishment of this Scheme is the only method which will provide them with the revenue essential for their continuation as voluntary institutions.

The Fund offers, in case of need, the following privileges to those contributors whose incomes are within the limits laid down by the British Medical Association :—

Free maintenance in voluntary hospitals on the recommendation of the panel or family doctor,

Grants for municipal hospital service,

Ambulance service,

Convalescent Home treatment, in accordance with the Fund's regulations.

Wherever possible, contributions (at the rate of 3d. a week) are deducted from salaries and wages by business houses and industrial establishments and forwarded to the Fund either direct or through any bank.

Employers are invited to add a penny in respect of each employee each week as a consolidated subscription to the six voluntary hospitals.

Servants in households where the domestic staff numbers less than five, assistants in shops where there are less than five employees, persons of small independent means, persons engaged in small businesses on their own account, etc., can contribute to the Fund by purchasing weekly hospital stamps from voluntary depot holders, namely, chemists, newsagents, etc.

Full information will gladly be sent on application to the Secretary, Bristol Hospitals Fund, Royal London House, Queen Charlotte Street, Bristol, 1.

